# Enhanced extracellular production of recombinant proteins in *Escherichia coli* by co-expression with *Bacillus cereus* phospholipase C

**DOI:** 10.1186/s12934-017-0639-3

**Published:** 2017-02-08

**Authors:** Lingqia Su, Qi Jiang, Lingang Yu, Jing Wu

**Affiliations:** 1 State Key Laboratory of Food Science and Technology, Jiangnan University, 1800 Lihu Avenue, Wuxi, 214122 China; 2 School of Biotechnology and Key Laboratory of Industrial Biotechnology Ministry of Education, Jiangnan University, 1800 Lihu Avenue, Wuxi, 214122 China

**Keywords:** *Bacillus cereus* phospholipase C, Co-expression, Extracellular protein production, Membrane permeability, Foam formation

## Abstract

**Background:**

Our laboratory has reported a strategy for improving the extracellular production of recombinant proteins through co-expression with *Thermobifida fusca* cutinase, which increases membrane permeability via its phospholipid hydrolysis activity. However, the foam generated by the lysophospholipid product makes the fermentation process difficult to control in a fermentor. Phospholipase C (PLC) catalyzes the hydrolysis of phospholipids to produce sn1,2-diacylglycerides and organic phosphate, which do not induce foam formation. Therefore, co-expression with *Bacillus cereus* PLC was investigated as a method to improve the extracellular production of recombinant proteins.

**Results:**

When *B. cereus* PLC was expressed in *Escherichia coli* without its signal peptide, 95.3% of the total PLC activity was detected in the culture supernatant. PLC expression enhanced membrane permeability without obvious cell lysis. Then, six test enzymes, three secretory and three cytosolic, were co-expressed with *B. cereus* PLC. The enhancement of extracellular production correlated strongly with the molecular mass of the test enzyme. Extracellular production of *Streptomyces* sp. FA1 xylanase (43 kDa), which had the lowest molecular mass among the secretory enzymes, was 4.0-fold that of its individual expression control. Extracellular production of glutamate decarboxylase (51 kDa), which had the lowest molecular mass among the cytosolic enzymes, reached 26.7 U/mL; 88.3% of the total activity produced. This strategy was effectively scaled up using a 3-L fermentor. No obvious foam was generated during this fermentation process.

**Conclusions:**

This is the first study to detail the enhanced extracellular production of recombinant proteins through co-expression with PLC. This new strategy, which is especially appropriate for lower molecular mass proteins, allows large-scale protein production in an easily controlled fermentation process.

## Background

*Escherichia coli* is the most widely studied and frequently used host for recombinant protein production because its genetics are well-characterized, the culture periods are short, and protein production levels are high [1–3]. Because *E. coli* has both inner and outer membranes, recombinant proteins produced in this host can localize to the cytoplasm, the periplasm, or the culture medium [2, 4]. Production of recombinant proteins in the culture medium is the most desirable of these alternatives, whether the protein is produced on an analytical or industrial scale, because the protein being produced experiences superior protein folding, requires a simpler extraction procedure, and is contaminated with fewer proteins [1, 5]. Unfortunately, *E. coli* is a relatively poor secretor of proteins, which is a significant barrier to the application to this methodology [6].

Many attempts have been made to enhance the extracellular production of recombinant secretory proteins in *E. coli*, and some success has been achieved [2, 6]. Two different approaches have been pursued. In one approach, protein secretion was enhanced through co-expression or modification of transport proteins [7–10]. The validity and universality of this approach may be compromised by the complexity and regulatory mechanisms associated with protein secretion systems. The other approach was to enhance nonspecific leakage of the recombinant proteins through the host cell membranes by constructing hosts with a leaky phenotype. Induction of the leaky phenotype can be accomplished through mutation or deletion of membrane components, addition of permeability enhancers (glycine, calcium, Triton X-100, etc.), or co-expression of proteins with lytic activity (bacterial phage lysis proteins) [2, 11–14]. Because the cell membrane is compromised to some extent, this approach has the inherent risks of impaired cell growth and cell lysis. Cytosolic proteins are generally expressed in the cytoplasm and extracted, after cell disruption, using physical, chemical, or biological methods. These processes result in contamination of the recombinant protein by various cellular components. Removing them requires costly down-stream processing [15–17].

In our previous study, *Thermobifida fusca* cutinase was shown to hydrolyze phospholipids. When this cutinase was expressed without an appended signal peptide in *E. coli* BL21(DE3), the permeability of the cell membrane was be enhanced due to limited hydrolysis of the phospholipid component [18]. This enhanced permeability efficiently improved the extracellular production of target proteins co-expressed with *T. fusca* cutinase, and no obvious cell lysis was seen during this process [19]. However, a large amount of foam that had an adverse effect on fermentation control was produced when this expression system was scaled up in 3-L fermentors. Further study showed that *T. fusca* cutinase catalyzed the hydrolysis of both of the phospholipid acyl groups, indicating the enzyme has phospholipase B activity (unpublished data). One of the hydrolysis products is lysophospholipid [20], a type of surfactant that readily induces the formation of foam.

Phospholipase C (PLC) catalyzes the cleavage of the glycerophosphate bond of phospholipids to produce *sn*1,2-diacylglycerides and organic phosphate [21]. These products should not induce the formation of foam. In this study, the PLC from *Bacillus cereus* was used to determine if PLC expressed in *E. coli* also enhances the permeability of the cell membrane. Six different test proteins, three secretory and three cytosolic, with different molecular masses were co-expressed with *B. cereus* PLC. The extracellular production of these test proteins was investigated in detail.

## Methods

### Bacterial strains, vectors and materials

*Bacillus cereus* and *Lactobacillus brevis* were purchased from the Microbial Resource Data Platform (Jiangnan University, Wuxi, China). *T. fusca* was taken from our laboratory stock. The construction of plasmids pET20b/*BgaD*-*D,* pET20b/*cgt,* and pET20b/*xynA*, pET24a/*Tfu_1891*, pET24a*/tres*, which harbor genes encoding *Bacillus circulans* β-galactosidase, *Paenibacillus macerans* α-cyclodextrin glucanotransferase (CGTase), *Streptomyces* sp. FA1 xylanase, *T. fusca* isoamylase, and *T. fusca* trehalose synthase, respectively, in our laboratory has been described [22–24]. *E. coli* strains JM109, BL21(DE3), and pETDuet-1 were purchased from Novagen (Madison, WI, USA). The plasmid vector pMD18-T simple, DNA polymerase, alkaline phosphatase, various restriction enzymes, T_4_ DNA ligase, and agarose were obtained from Takara (Dalian, China). Genomic DNA, plasmid preparation kits and agarose gel DNA purification kits were purchased from Tiangen Biotech Co., Ltd (Beijing, China). Primer synthesis and DNA sequencing were performed by Shanghai Generay Biotech Co. Ltd (Shanghai, China). *O*-Nitrophenyl β-d-galactopyranoside (*o*NPG), beechwood xylan, 1,2-phthalic dicarboxaldehyde, pyridoxal 5′-phosphate (PLP), and γ-aminobutyric acid were obtained from Sigma (Shanghai, China). Other reagents were from Sinopharm Chemical Reagent Co., Ltd. (Shanghai, China).

### Construction of *B. cereus* PLC expression plasmid

The gene encoding *B. cereus* PLC (*plc*) was amplified from the *B. cereus* genome using PCR with primers 5′-CCATGGGCCATGAAAATGATGGGGGAAG-3′ and 5′-AAGCTTAACGATCTCCGTACGTATC-3′. The underlined sequences represent *Nco*I and *Hin*dIII restriction sites, respectively. The PCR product was ligated into the plasmid vector pMD18-T simple, and its sequence was confirmed by DNA sequencing. The *plc* gene was excised from the pMD18-T derivative using restriction endonucleases *Nco*I and *Hin*dIII, and then ligated into similarly restricted plasmid pETDuet-1, yielding plasmid pETDuet/*plc*. This procedure placed *plc* in the first multiple cloning sequence of the pETDuet vector.

The mutant plasmid pETDuet/*plcD55L* was generated from pETDuet/*plc* using standard QuikChange mutagenesis methodology and the primers 5′-GTATTTATGCTGCTCTGTATGAAAATCCTTATTATGA-3′ and 5′-TAAGGATTTTCATACAGAGCAGCATAAATACCGTTCT-3′. The mutant sequence was confirmed by DNA sequencing.

### Construction of plasmids for the expression of secretory enzymes

The gene encoding *B. circulans* β-galactosidase (*BgaD*-*D*), including its pelB signal peptide, which was used to induce secretory expression of the enzyme, was excised from the previously constructed plasmid pET-20b/*BgaD*-*D* using restriction endonucleases *Nde*I and *Xho*I. The resulting fragment was ligated into the second multiple cloning site of similarly restricted pETDuet-1 or pETDuet/*plc*, resulting in the two recombinant plasmids, pETDuet/*BgaD*-*D* and pETDuet/*plc*/*BgaD*-*D*, which direct the expression of β-galactosidase alone and co-expression of PLC and β-galactosidase, respectively. This approach was also used to construct plasmids pETDuet/*cgt*, pETDuet/*plc*/*cgt*, pETDuet/*xynA* and pETDuet/*plc*/*xynA*, which direct the expression of *P. macerans* CGTase and *Streptomyces* sp. FA1 xylanase alone, and for their co-expression with PLC, respectively.

### Construction of plasmids for the expression of cytosolic enzymes

The gene encoding *T. fusca* isoamylase (*Tfu_1891*) was excised from the previously constructed plasmid pET24a/*Tfu_1891* using restriction endonucleases *Nde*I and *Xho*I [24], and then ligated into the second multiple cloning site of pETDuet-1 or pETDuet/*plc* to construct the plasmids pETDuet/*Tfu_1891* and pETDuet/*plc*/*Tfu_1891*. These plasmids direct the expression of isoamylase alone and the co-expression of isoamylase with PLC, respectively. A similar approach was used to construct plasmids pETDuet/*tres*, pETDuet/*plc*/*tres*, pETDuet/*gad*, and pETDuet/*plc*/*gad,* which direct the expression of *T. fusca* trehalose synthase and *L. brevis* glutamate decarboxylase; alone and co-expressed with PLC, respectively.

### Cultivation conditions

The plasmids described above were used to transform chemically competent *E. coli* BL21(DE3) cells. These engineered *E. coli* strains were cultured in shake flasks or in a 3-L fermentor to produce the test proteins.

The shake-flask fermentation was performed as follows. A 10-mL aliquot of Luria–Bertani medium (g/L: tryptone, 10.0; yeast extract, 5.0; NaCl, 10.0) supplemented with 100 μg/mL ampicillin was inoculated with a frozen glycerol stock of the engineered *E. coli* strain (20 μL), and then incubated for up to 8 h in a rotary shaker (200 rpm) at 37 °C. An aliquot of this seed culture (5% [v/v]) was used to inoculate 50 mL of Terrific Broth medium (g/L: glycerol, 5.0; tryptone, 12.0; yeast extract, 24.0; KH_2_PO_4_, 2.3; and K_2_HPO_4_, 16.4) supplemented with 100 μg/mL ampicillin, which was then shaken in a rotary shaker (200 rpm) at 37 °C. When the culture’s optical density at 600 nm (OD_600_) increased to 1.5, the inducer isopropyl β-d-thiogalactopyranoside (IPTG) was added to a final concentration of 0.4 mM and incubation was continued at 25 °C. Samples of each culture were collected and analyzed for biomass and enzyme activities at designated time intervals.

Fed-batch cultivation of the engineered *E. coli* was performed in a 3-L fermentor (Labfors 5; Infors-HT Co., Ltd) using a previously described two-phase feeding strategy [25]. The seed culture was prepared as described in the previous section, and then used to inoculate an initial batch of semisynthetic medium (g/L: glycerol, 8.0; tryptone, 30.0; yeast extract, 20.0; K_2_HPO_4_, 14.6; MgSO_4_·7H_2_O, 2.0; (NH_4_)_2_-H-citrate, 1.2; and trace metal solution, 1.0 mL/L [25]) supplemented with 100 μg/mL ampicillin. When the initial glycerol was completely consumed, which was detected by the sudden increase in dissolved oxygen and pH, the culture was continuously fed using the feeding solution (g/L: glycerol, 500.0; tryptone, 50.0; yeast extract, 50.0; and MgSO_4_·7H_2_O, 3.4). When the OD_600_ of the culture reached 50, lactose was added to the fermentation at 0.2 g/L/h to induce protein expression. The temperature kept at 37 °C during the growth phase and 30 °C during the induction phase. The pH of the fermentation was 7.0, and the dissolved oxygen level was approximately 30% of air saturation. The biomass and enzyme activities were analyzed at designated time intervals.

### Determination of biomass

The OD_600_ of the culture was measured using a spectrophotometer. If the OD_600_ value was higher than 0.8, samples were diluted with 0.9% (w/v) NaCl solution to an OD_600_ value of 0.2–0.8. To determine the dry cell weight (DCW), a 10-mL aliquot of the culture broth was centrifuged at 12,000×*g* for 10 min. The resulting pellet was collected and washed with 0.9% (w/v) NaCl solution twice, then dried at 105 °C until the weight remained constant.

### Determination of PLC and test enzyme activities

PLC activity was determined using *p*-nitrophenylphsophorylcholine (*p*-NPPC) as the substrate. The reaction mixture contained Tris–HCl buffer (50 mM, pH 7.2), 0.1 mM ZnCl_2_, 10 mM *p*-NPPC, and 20 μL of the appropriately diluted enzyme. After incubation at 37 °C for 10 min, the optical density of the reaction mixture at 410 nm was determined. One unit of PLC activity was defined as the amount of enzyme that produced 1 nmol *p*-nitrophenol (*p*NP) per min under the conditions described above.

β-Galactosidase activity was determined by measuring the conversion of *o*NPG into *o*-nitrophenol. The reaction was started by adding the appropriately diluted enzyme (0.1 mL) into sodium phosphate buffer (50 mM, pH 6.5; 1.9 mL) containing 20 mM *o*NPG. After incubation at 50 °C for 10 min, 1 mL of 1 M Na_2_CO_3_ was added to stop the reaction. The absorbance of the reaction mixture at 420 nm was measured. One unit of β-galactosidase activity was defined as the amount of enzyme needed to produce 1 μmol *o*-nitrophenol per min.

CGTase activity was determined by measuring its α-cyclodextrin forming activity. A reaction mixture consisting of 0.9 mL 3% (w/v) soluble starch in phosphate buffer (50 mM, pH 6.0) and 0.1 mL appropriately diluted enzyme was incubated at 40 °C for 10 min. The reaction was quenched by adding 1 mL of HCl (1.0 M). After that, 1.0 mL of methyl orange (0.1 mM) was added, and the mixture was incubated at 16 °C for 20 min for color development. The amount of α-cyclodextrin produced was determined by measuring the optical density at 505 nm. One unit of CGTase activity was defined as the amount of enzyme that produced 1 μmol of α-cyclodextrin per min.

Xylanase activity was determined by measuring the release of reducing sugar using beechwood xylan as the substrate. The reaction mixture, which contained 1% (w/v) beechwood xylan and appropriately diluted enzyme in sodium phosphate buffer (50 mM, pH 5.5), was incubated at 50 °C for 10 min. The amount of reducing sugar released was measured as previously reported [26]. One unit of xylanase activity was defined as the amount of enzyme needed to produce 1 μmol of xylose per min.

Isoamylase activity was determined by measuring the hydrolysis of amylopectin in a reaction mixture containing 3 mL waxy corn amylopectin (0.83%) in Na_2_HPO_4_- citric acid (50 mM, pH 5.5) buffer and 0.5 mL appropriately diluted enzyme. The reaction was performed at 50 °C for 30 min. A 0.5 mL aliquot of the reaction mixture was added to 0.5 mL of 10 mM I_2_–0.1 M KI solution and 15 mL of 20 mM sulfuric acid. The color was allowed to develop at room temperature for 10 min, and the absorbance at 610 nm was measured. One unit of isoamylase activity was defined as the amount of enzyme needed to cause an absorbance increase of 0.01 per min.

Trehalose synthase activity was determined by measuring the conversion of maltose to trehalose. A reaction mixture containing 400 µL of 5% (w/v) maltose in sodium phosphate buffer (20 mM, pH 7.0) and 400 µL of appropriately diluted enzyme was incubated at 30 °C for 30 min, and then boiled for 10 min. The amount of trehalose generated during the reaction period was detected using an Agilent 1200 HPLC system (Agilent Technologies, Palo Alto, CA, USA). One unit of trehalose synthase activity was defined as the amount of enzyme that produced 1 μmol of trehalose per min.

Glutamate decarboxylase activity was determined by measuring the generation of γ-aminobutyric acid (GABA) from glutamic acid in a reaction mixture containing 50 mM citric acid-Na_2_HPO_4_ buffer (pH 4.8), 100 mM glutamic acid, 0.15 mM PLP, and appropriately diluted enzyme. The reaction mixture was incubated at 37 °C for 4 min, and then the reaction was terminated by adding 0.6 mL borate (0.2 M, pH 10.0), and boiling for 10 min. The GABA in the reaction mixture was quantitated using an Agilent 1200 HPLC system (Agilent Technologies, Palo Alto, CA, USA). One unit of glutamate decarboxylase activity is defined as the amount of enzyme that produced 1 μmol GABA per min.

### Assay of membrane permeability

Inner membrane permeability was determined by measuring the ability of *o*NPG to access the cytoplasm. *o*NPG was added to the cell suspension (OD_600_ 0.15) at a final concentration of 100 μg/mL. The absorption of light at 420 nm was monitored using a spectrophotometer (Shimadzu Co., Japan).

Outer membrane permeability was determined using the 1-N-phenylnaphthylamine (NPN) access assay. NPN was added to the cell suspension (OD_600_ 0.15) at a final concentration of 10 μM. The fluorescence of the mixture was monitored using a Shimadzu RF-1501 spectrofluorometer (Shimadzu Co., Japan) with slit widths of 5 nm and excitation and emission wave lengths of 350 and 420 nm, respectively.

### Determination of cell lysis

Cell lysis was quantified as the percentage of extracellular β-galactosidase activity, as previously described [27]. Briefly, the extracellular β-galactosidase activity, determined using an established method [28], was divided by the sum of intracellular and extracellular β-galactosidase activities. The resulting fraction was multiplied by 100 to provide a percentage.

### Transmission electron microscopy (TEM)

Engineered *E. coli* BL21(DE3) were centrifuged at 4000×*g* for 2 min. The cell pellets were fixed in 2.5% glutaraldehyde, rinsed 3 times with sodium phosphate buffer (0.1 M, pH 7.2–7.4), post-fixed in 1% osmic acid, and then rinsed again. After that, the cells were dehydrated using ethyl alcohol and embedded in araldite. The sample was cut into 70-nm-thick slices, stained with uranyl acetate and lead citrate, and examined using a JEM-7000 transmission electron microscope (JEOL, Japan). The TEM analysis was performed by The Biomedical Research Center of Zhongshan Hospital, Fudan University (Shanghai, China).

## Results and discussion

### Expression of *B. cereus* PLC in *E. coli* BL21(DE3)

**Fig. 1:**
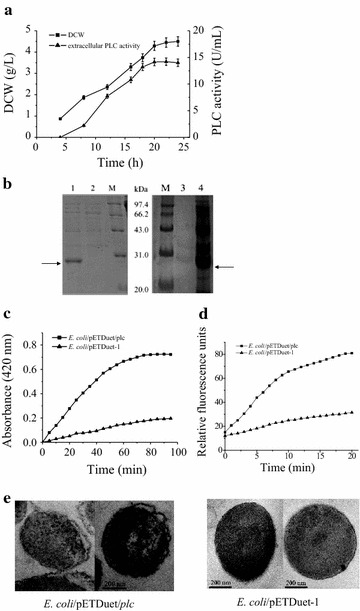
Expression of *B. cereus* PLC in *E. coli* BL21(DE3). **a** Kinetics of DCW and extracellular PLC activity. *Error bars* represent the standard deviation from three replicate measurements. **b** SDS-PAGE analysis of the culture supernatants of *E. coli*/pETDuet/*plc* (*lane 1*) and *E. coli*/pETDuet-1 (*lane 2*), as well as the culture supernatant (*lane 3*) and intracellular fraction (*lane 4*) of *E. coli*/pETDuet/*plcD55L*. **c** Inner membrane permeability of *E. coli*. **d** Outer membrane permeability of *E. coli*. **e** TEM-images of *E. coli*

The gene encoding PLC was cloned from *B. cereus* genomic DNA using PCR and inserted into the expression plasmid pETDuet-1, placing the gene under the control of the first T7 promoter. This recombinant plasmid, pETDuet-1/*plc*, was used to transform *E. coli* BL21(DE3). When the modified *E. coli* strain was cultivated in shake flasks, no PLC activity was detected in the culture supernatant after the first 4 h of cultivation. After 4 h, the PLC activity in the supernatant gradually increased, reaching 14.2 U/mL at 20 h. During this time, intracellular PLC activity remained at only 0.7 U/mL. Thus, 95.3% of the target PLC was released into the culture supernatant (Fig. 1a). SDS-PAGE analysis of the culture supernatant revealed a single major band around 28 kDa, which is consistent with the calculated molecular mass of PLC (Fig. 1b). No PLC activity was detected in either the intracellular or extracellular fractions of a control *E. coli* BL21(DE3) strain that contained the parent vector pETDuet-1.

Proteins lacking a signal peptide are generally unable to cross cell membranes; therefore, they are located in the cytoplasm [24, 29, 30]. The results described above show that when PLC lacking a signal peptide is expressed in *E. coli*, the PLC is released into the culture medium. The relationship between this phenomenon and PLC’s phospholipase activity was examined by expressing a PLC mutant with almost no activity. It has been reported that Asp55 plays a critical role in catalytic activity and that mutant D55L retains only 0.00009% of the activity of wild-type PLC [31]. In this study, the mutant D55L was expressed under conditions similar to those of PLC. All of the mutant protein was localized within the cell (Fig. 1b). Further study showed that *E. coli* expressing PLC also showed significantly increased inner and outer membrane permeability, the ability of substances to non-specifically cross the membranes, compared with the control, based on *o*NPG and NPN access assay, respectively (Fig. 1c, d). Similar results were obtained when *T. fusca* cutinase lacking a signal peptide was expressed in *E. coli* [18]. This suggests that PLC, which possesses phospholipase activity, is released into the culture medium through nonspecific cell leakage induced by the hydrolysis of membrane phospholipid, as was seen with *T. fusca* cutinase [18]. Further experiments were performed, using the endogenous cytoplasmic β-galactosidase as a reporter protein, to determine if PLC caused cell lysis [27]. Our results showed that the percentage of β-galactosidase activity in the culture medium at the time when the extracellular PLC activity had reached its highest value (20 h post-induction) was 6.9%; similar to that (6.5%) of control cells harboring the vector pETDuet-1. This demonstrates that *E. coli* BL21(DE3) cells expressing recombinant protein are not prone to lysis. In order to further observe the effect of PLC expression on cell morphology, *E. coli* expressing PLC and control cells were subjected to TEM analysis. The results showed that *E. coli* cells expressing PLC exhibited an irregular shape with a distorted cell membrane, compared with the control cells, but remained intact (Fig. 1e). Thus, cells expressing PLC suffered limited damage that could cause the release of substances inside the cell, but no obvious cell lysis occurred.

To investigate if the limited damage to the cell membranes caused by the expression of *B. cereus* PLC could enhance the extracellular production of other recombinant proteins, six different test proteins, including three secretory and three cytosolic enzymes, were co-expressed with *B. cereus* PLC. Because our hypothesis was that enhanced extracellular production was dependent on nonspecific cell leakage and the efficiency of this process was expected to be dependent on the test proteins’ molecular masses, proteins with different molecular masses were selected.

### Co-expression of secretory enzymes with *B. cereus* PLC

Three secretory enzymes, *B. circulans* β-galactosidase (86 kDa), *P. macerans* CGTase (76 kDa), and *Streptomyces* sp. FA1 xylanase (43 kDa), were selected as the model proteins. These proteins have the potential for wide application [22, 23].

### Co-expression of *B. circulans* β-galactosidase with *B. cereus* PLC

The gene (*BgaD*-*D*) encoding *B. circulans* β-galactosidase with an attached pelB signal peptide was inserted into pETDuet-1, and pETDuet-1/*plc*, respectively, placing *BgaD*-*D* under the control of the second T7 promoter. These recombinant plasmids were inserted into *E. coli* BL21(DE3), yielding separate strains for the expression β-galactosidase alone and the co-expression of β-galactosidase with PLC. When cultivated in shake flasks, the highest β-galactosidase activities in the culture supernatant were 18.9 U/mL at 50 h (β-galactosidase alone) and 15.1 U/mL at 45 h (co-expression). The productivity of the co-expression system was only 89.2% that of the control system. β-Galactosidase production was clearly not enhanced by co-expression with *B. cereus* PLC.

### Co-expression of *P. macerans* CGTase with *B. cereus* PLC

**Fig. 2:**
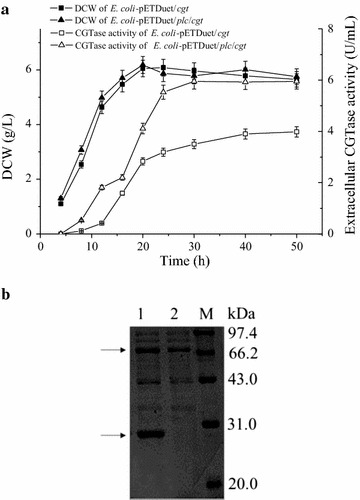
Expression of CGTase in *E. coli* BL21(DE3). **a** Kinetics of DCW and extracellular CGTase activity. *Error bars* represent the standard deviation from three replicate measurements. **b** SDS-PAGE analysis of the culture supernatants of *E. coli*/pETDuet/*plc*/*cgt* (*lane 1*) and *E. coli*/pETDue*t*/*cgt* (*lane 2*)

*Escherichia coli* BL21(DE3) strains suitable for the expression of the CGTase alone and the co-expression of the CGTase with *B. cereus* PLC were constructed using the approach described above for β-galactosidase. When cultivated in shake flasks, the two strains grew similarly. The CGTase activity in the culture supernatant of the *E. coli* cells expressing CGTase alone gradually increased and reached a maximum of 3.98 U/mL at 50 h. When CGTase was co-expressed with PLC, more activity (5.95 U/mL) was obtained after a shorter period of time (30 h). This equates to a productivity of 0.2 U/mL/h, which is 2.5 fold that of *E. coli* cells expressing CGTase alone (Fig. 2a). SDS-PAGE analysis also showed an apparent protein band corresponding to the molecular mass of CGTase (Fig. 2b).

### Co-expression of *Streptomyces* sp. FA1 xylanase with *B. cereus* PLC

**Fig. 3:**
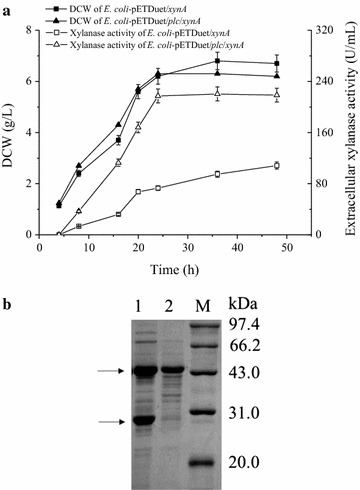
Expression of xylanase in *E. coli* BL21(DE3). **a** Kinetics of DCW and extracellular xylanase activity. *Error bars* represent the standard deviation from three replicate measurements. **b** SDS-PAGE analysis of the culture supernatants of *E. coli*/pETDuet/*plc*/*xynA* (*lane 1*) and *E. coli*/pETDue*t*/*xynA* (*lane 2*)

*Escherichia coli* BL21(DE3) strains suitable for the expression of *Streptomyces* sp. FA1 xylanase alone and the co-expression of this xylanase with *B. cereus* PLC were constructed as described for *B. circulans* β-galactosidase. When they were cultivated in shake flasks, the growth curves of the *E. coli* strains were similar. The highest DCW of cells expressing xylanase alone was 8% higher than that of cells co-expressing xylanase and PLC. The maximum xylanase activities in their culture supernatants were 108.3 U/mL at 48 h (xylanase alone) and 217.2 U/mL at 24 h (co-expression). Thus, the extracellular productivity of the co-expression strain (9.1 U/mL/h) was 4.0 times higher than that of the control (2.3 U/mL/h) (Fig. 3a). The successful extracellular production of xylanase was also verified using SDS-PAGE analysis (Fig. 3b).

Taken together, these results show that the enhancement of extracellular yield gained by co-expression with *B. cereus* PLC increased as the molecular mass of the test protein decreased. This is precisely the result expected when the mechanism behind the enhancement is limited nonspecific cell leakage. In this mechanism, PLC catalyzed the hydrolysis of membrane phospholipids, which weakened the cell membrane and enhanced membrane permeability. When this occurred, the target protein was more efficiently secreted into the culture medium than it was in the absence of co-expression. This improved protein production would provide the benefits of maintaining a balance with the high expression level, which is of critical importance for the extracellular production of recombinant proteins [32, 33]. The lack of enhancement seen with the high-molecular-weight protein *B. circulans* β-galactosidase is consistent with the nonspecific cell leakage mechanism, since the leakage of large proteins is expected to be extremely inefficient. It also provides further evidence that cell lysis is not a substantial contributor, since enhanced lysis is expected to enhance the extracellular production of all proteins, regardless of their molecular mass.

When the secretory proteins were co-expressed with PLC, the pelB signal peptide was attached to the N-termini of the secretory proteins. Thus, it is likely that some of the protein was secreted via the Sec pathway and some via the PLC mechanism. To investigate whether the signal peptide was removed in both cases, N-terminal sequencing was conducted on the extracellular xylanase, whose production was enhanced most significantly among the secretory enzymes. The results showed that the first five amino acids of the N-terminus were MAENT, which match the N-terminal residues of mature xylanase. Thus, the signal peptide could be removed after xylanase crossed through the cell membranes.

### Extracellular production of *Streptomyces* sp. FA1 xylanase in 3-L fermentor

The expression of *Streptomyces* sp. FA1 xylanase, which had the lowest molecular mass among the secretory proteins tested, was scaled up in a 3-L fermentor. *E. coli* BL21(DE3) strains expressing xylanase alone and co-expressing xylanase with PLC were each cultured using a fed-batch method to achieve high cell density. Cell growth and xylanase production were monitored.

**Fig. 4:**
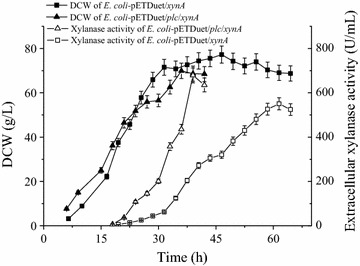
Extracellular production of xylanase in a 3-L fermentor. *Error bars* represent the standard deviation from three replicate measurements

Figure 4 shows that *E. coli* expressing xylanase alone grew well. The highest DCW was 77.2 g/L at 46.5 h, with a slight decrease occurring during the later period of fermentation. The extracellular xylanase activity gradually increased after induction, and reached 550.2 U/mL at 61.5 h (45 h after induction), which equated to a productivity of 12.1 U/mL/h. At this time, the intracellular xylanase activity was 183.4 U/mL; thus, 75% of the total xylanase activity was secreted into the culture medium. The growth of *E. coli* BL21(DE3) cells co-expressing xylanase with PLC was similar to that of *E. coli* BL21(DE3) expressing xylanase alone for the first 21 h of fermentation (6 h after induction). After that, the increase in DCW was a little slower than that observed with xylanase alone, probably because of PLC-mediated damage of the cell membrane. The highest DCW observed during co-expression was 69.9 g/L at 36 h. The peak value of the extracellular xylanase activity was 678.6 U/mL at 39 h (24 h after induction), which accounted for 80% of the total xylanase activity. The productivity seen with co-expression (21.7 U/mL/h) was 1.8-fold that seen with *E. coli* BL21(DE3) expressing xylanase alone, indicating a potential advantage for the co-expression of xylanase and *B. cereus* PLC in a 3-L fermentor (Fig. 4).

PLC cleaves phospholipids into diacylglycerides and phosphate monoesters [20, 21], neither of which were expected to cause the formation of foam. As expected, no obvious foam was produced during co-expression, which makes this fermentation easy to control and especially suitable for industrial application.

### Co-expression of *B. cereus* PLC with cytosolic enzymes

Three cytosolic enzymes, *T. fusca* isoamylase (79 kDa), *T. fusca* trehalose synthase (66 kDa), and *L. brevis* glutamate decarboxylase (53 kDa) were also selected as test proteins. These proteins also have the potential for wide application [24, 34, 35].

### Co-expression of *T. fusca* isoamylase with *B. cereus* PLC

The gene encoding *T. fusca* isoamylase was inserted into pETDuet-1 and into pETDuet/*plc*, resulting in plasmids that direct the expression of isoamylase alone and co-expression of the isoamylase with PLC, respectively. The plasmids were inserted into *E. coli* BL21(DE3), and the engineered *E. coli* strains were cultivated in shake flasks for 30 h. Investigation of the isoamylase activity in different cell fractions showed that the isoamylase was always located in the cytoplasmic fraction; no isoamylase activity was detected in the culture medium of either expression strain. This result is similar to that obtained with *B. circulans* β-galactosidase, and is entirely consistent with the model in which *B. cereus* PLC enhances the extracellular production of co-expressed proteins by increasing membrane permeability and the enhancement is dependent upon the molecular mass of the test protein. It is also consistent with the idea that *B. cereus* PLC does not enhance cell lysis, because lysis would allow all of cellular substances to be release into the culture medium.

### Co-expression of *T. fusca* trehalose synthase with *B. cereus* PLC

**Fig. 5:**
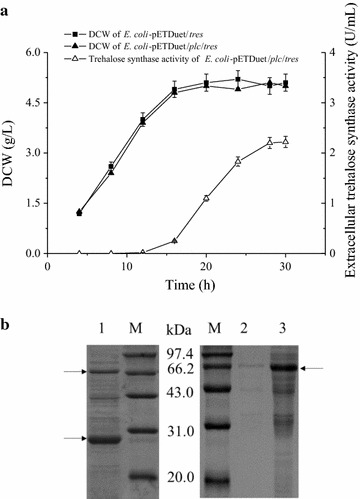
Expression of trehalose synthase in *E. coli* BL21(DE3). **a** Kinetics of DCW and extracellular trehalose synthase activity. *Error bars* represent the standard deviation from three replicate measurements. **b** SDS-PAGE analysis of the culture supernatant of *E. coli*/pETDuet/*plc*/*tres* (*lane 1*), as well as the culture supernatant (*lane 2*) and intracellular fraction (*lane 3*) of *E. coli*/pETDue*t*/*tres*

Recombinant *E. coli* strains for the expression of *T. fusca* trehalose synthase alone and for the co-expression of *T. fusca* trehalose synthase with *B. cereus* PLC were constructed using the approach described in the last section for *T. fusca* isoamylase. The two *E. coli* strains exhibited similar growth characteristics in shake flasks. With the co-expression system, trehalose synthase activity in the culture medium began to gradually increase after 12 h of cultivation, and reached its peak value of 2.2 U/mL at 28 h (Fig. 5a). This activity accounted for 35.2% of the total activity in all of the cell fractions. Thus, co-expression with *B. cereus* PLC allowed about one-third of the cytosolic enzyme *T. fusca* trehalose synthase, which has a smaller molecule weight than *T. fusca* isoamylase, to be release into the culture medium. In addition, when expressed alone, no trehalose synthase activity was detected in the culture medium at any time during the fermentation period, and the trehalose synthase expressed alone remained in the cytoplasm (Fig. 5b).

### Co-expression of *L. brevis* glutamate decarboxylase with *B. cereus* PLC

**Fig. 6:**
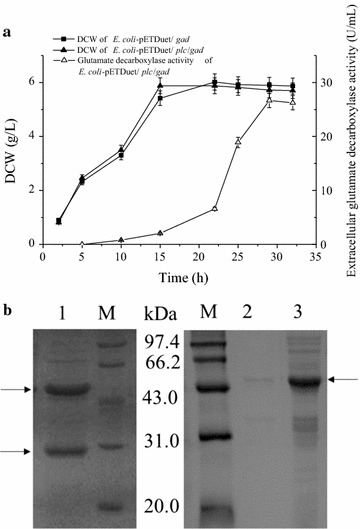
Expression of glutamate decarboxylase in *E. coli* BL21(DE3). **a** Kinetics of DCW and extracellular glutamate decarboxylase activity. *Error bars* represent the standard deviation from three replicate measurements. **b** SDS-PAGE analysis of the culture supernatant of *E. coli*/pETDuet/*plc*/*gad* (*lane 1*), as well as the culture supernatant (*lane 2*) and intracellular fraction (*lane 3*) of *E. coli*/pETDue*t*/*gad*

Recombinant *E. coli* strains for the expression of *L. brevis* glutamate decarboxylase alone and for the co-expression of *L. brevis* glutamate decarboxylase with *B. cereus* PLC were constructed using the approach for *T. fusca* isoamylase. The two *E. coli* strains were then cultivated in shake flasks. Both of the strains grew well. When co-expressed with *B. cereus* PLC, the extracellular glutamate decarboxylase activity reached its highest value, 26.7 U/mL, at 29 h (Fig. 6a). This represented 88.3% of the total activity. When glutamate decarboxylase was expressed alone, no glutamate decarboxylase activity was detected in culture medium, and SDS-PAGE analysis of glutamate decarboxylase expressed alone showed that the glutamate decarboxylase was located in the cytoplasm, as expected (Fig. 6b).

Extracellular production of cytosolic enzymes is not expected in the presence of a fully functional cell membrane. Therefore, the extracellular production observed must have depended on protein leaking through the cell membrane. The efficiency of this extracellular production increased as the molecular mass of the cytosolic protein decreased. This result is similar to that observed with the secretory enzymes. Thus, co-expression with *B. cereus* PLC enhanced the extracellular production of both secretory and cytosolic enzymes, and the enhancement was strongly related to the molecular mass of the test protein. More detailed investigations surrounding the influence of test protein molecular mass and other potential factors on the enhancement of extracellular protein production are underway.

### Extracellular production of *L. brevis* glutamate decarboxylase in a 3-L fermentor

**Fig. 7:**
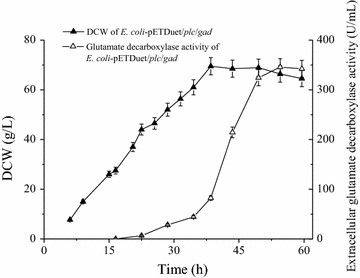
Extracellular production of glutamate decarboxylase in a 3-L fermentor. *Error bars* represent the standard deviation from three replicate measurements

The co-expression of *L. brevis* glutamate decarboxylase with *B. cereus* PLC was performed in a 3-L fermentor. The cells grew well, and expression of glutamate decarboxylase was induced by the continuous addition of lactose. The extracellular glutamate decarboxylase activity gradually increased during the induction period, and the final yield was 345.5 U/mL. This activity accounted for 72.0% of the activity observed in all of the cell fractions. The highest biomass attained was 69.5 g/L (Fig. 7).

Cytosolic target proteins have generally been extracted from disrupted cells, which have traditionally been lysed using physical or chemical methods [16, 29, 34, 35]. Thus far, the use of mild and eco-friendly biological approaches, such as co-expression with lysis proteins, has been fraught with concerns [15, 16, 36]. Because the cells were usually lysed, the target proteins were contaminated with a variety of cellular components, which led the costly down-stream processing. This study provides an effective and convenient approach for the extracellular production of cytosolic proteins without obvious cell lysis. In addition, there is no obvious foam production during the fermentation process, which is a benefit for control during large-scale production of industrial proteins.

## Conclusions

*Bacillus cereus* PLC expressed in *E. coli* without a signal peptide was released into the culture medium. This extracellular production was dependent on the increased membrane permeability caused by the limited hydrolysis of membrane phospholipids. The increased membrane permeability could efficiently enhance the extracellular release of the relatively low molecular mass secretory and cytosolic enzymes tested. This is the first report to provide a detailed description of the extracellular production of recombinant proteins through co-expression with PLC. The effective extracellular production of proteins and convenient process described here provide an attractive alternative to meet the demands of wholesale industrialization.
